# Stress, Time Pressure, Strategy Selection and Math Anxiety in Mathematics: A Review of the Literature

**DOI:** 10.3389/fpsyg.2017.01488

**Published:** 2017-09-01

**Authors:** Sara Caviola, Emma Carey, Irene C. Mammarella, Denes Szucs

**Affiliations:** ^1^Department of Psychology, Centre for Neuroscience in Education, University of Cambridge Cambridge, United Kingdom; ^2^Department of Developmental Psychology, University of Padua Padua, Italy

**Keywords:** time constraints, arithmetic, problem solving, strategies, stress, math anxiety

## Abstract

We review how stress induction, time pressure manipulations and math anxiety can interfere with or modulate selection of problem-solving strategies (henceforth “strategy selection”) in arithmetical tasks. Nineteen relevant articles were identified, which contain references to strategy selection and time limit (or time manipulations), with some also discussing emotional aspects in mathematical outcomes. Few of these take cognitive processes such as working memory or executive functions into consideration. We conclude that due to the sparsity of available literature our questions can only be partially answered and currently there is not much evidence of clear associations. We identify major gaps in knowledge and raise a series of open questions to guide further research.

## Introduction

Negative feelings and stressful situations can interfere to different degrees with success in mathematical tasks (Ashcraft and Kirk, 2001; Maloney and Beilock, 2012; Vukovic et al., 2013). Here, we systematically review the existing literature on the relationship between experiencing worrying/demanding situations (through stress induction), time pressure manipulations, math anxiety and strategy selection. Our main question is whether stressors in mathematics drive selection of more efficient strategies (i.e., providing the best accuracy within the constraints of the new situation) or whether they cause suboptimal strategy selection due to inducing interfering worrying thoughts.

Strategic behavior (i.e., deciding between two or more available options) is used in a wide range of problem solving domains, not only in the field of education (Pillutla and Murnighan, 1995; Hughes, 1998; Olthof et al., 2011). A wide body of research in the decision-making field states that individuals use a variety of strategies to make choices (Abelson and Levi, 1985). Similarly to in the arithmetic field, the selection of a particular strategy is contingent on many task- and context-related variables (Payne et al., 1988), suggesting that people can also adaptively change processing strategies appropriately when modest changes occur in the structure of the problems. Stress both influences and is influenced by strategy selection, resulting in quite a strong association between strategy selection and stress responses (Starcke and Brand, 2012).

In the mathematical domain, a wide body of research suggests that mathematics builds on several cognitive abilities (Passolunghi et al., 2008; Krajewski and Schneider, 2009; Geary, 2011) implemented by an extended neural network of the brain (Goswami and Szűcs, 2011; Fias et al., 2013; Szűcs et al., 2014). The flexibility and adaptability of strategic behavior coordinating these cognitive abilities (Verschaffel et al., 2009; Rittle-Johnson et al., 2012) is highly important as the correct execution of arithmetic problems typically involves a series of steps, which include adaptively switching between different arithmetic strategies in order to select and apply the most efficient one (Siegler and Shipley, 1995; Siegler and Lemaire, 1997). This choice can be influenced or driven by different factors. Some of these are linked to the features of the problem itself (such as the complexity of the algorithm in Imbo and LeFevre, 2010). Others are related to personal features of the solver in terms of both domain-specific aspects (such as their mathematical expertise and attitudes/emotions toward math; Baroody and Dowker, 2003) and domain-general aspects, i.e., broader cognitive and emotional factors (Devine et al., 2012; Mammarella et al., 2015).

Among cognitive factors, the process most widely explored and strongly related to the successful performance on arithmetical tasks is working memory (WM; for review see Raghubar et al., 2010; Bull and Lee, 2014; see also Passolunghi et al., 2008; Friso-Van Den Bos et al., 2013; Szűcs, 2016). WM is commonly analyzed as a predictor to explain mathematic outcomes at a later point in time, and the number of studies investigating this relationship has sharply increased in recent years (Bull et al., 2008; LeFevre et al., 2012; Li and Geary, 2013; Caviola et al., 2014; Cragg and Gilmore, 2014; Szűcs et al., 2014). This evidence supports the view that WM influences math achievement in different ways: it might help to keep track of relevant information (e.g., storage and retrieval of partial results) during a problem-solving process, as well as being involved in the successful selection and implementation of procedures (Barrouillet and Lépine, 2005; Swanson, 2006; Wu et al., 2008; Meyer et al., 2010).

While important for mathematical processes, WM is also highly sensitive to interference from stressors. Ashcraft and Kirk (2001) and Ashcraft and Krause (2007) found that cognitive processes can be negatively affected by the interference of negative emotions, such as math anxiety or pressured situations. This suggests how negative feelings might overload the WM system, thus resulting in a drop in performance (e.g., failing to achieve the result due the application of an inefficient strategy). Eysenck and colleagues tried to define the interaction between negative feelings and WM by developing the Attentional Control Theory (ACT, Eysenck and Calvo, 1992; Eysenck and Derakshan, 2011). According to this theory, anxiety affects participants' performance by disrupting their ability to control attention, making them more susceptible to distraction. This theory postulates that anxiety shifts the attention to task irrelevant stimuli by reducing cognitive resources allocated to the concurrent (relevant) task. This mechanism damages the subject's efficiency, whether the distracting stimuli are external (i.e., task-irrelevant stimuli) or internal (i.e., worrying thoughts; self-preoccupation). In summary, according to this model, anxiety affects the central executive component of WM processes, leading to a reduced cognitive performance in terms of decreased task efficiency and effectiveness, particularly on complex tasks (Ramirez and Beilock, 2011; Mammarella et al., 2017). Another important determinant of mathematical strategy selection is problem difficulty or complexity. The likelihood of choosing one strategy rather than another varies with problem features (Siegler, 1996; Lemaire and Callies, 2009). Increasing problem difficulty promotes the use of more advanced computational strategies, in order to maximize efficiency while still maintaining accuracy. The complexity of a problem can be manipulated in different ways (e.g., the type of algorithm, the number of digits in the operands, the presence or absence of a carrying procedure, etc.) resulting in different WM demands (Imbo and LeFevre, 2010). In fact, increasing the complexity of a problem can itself act as a stressor or modulate the effect of stress on math task execution. Efficiency usually decreases when carry or borrow problems have to be performed due to an increase in WM demand (Noël et al., 2001; Imbo et al., 2007; Caviola et al., 2012). Previous studies have also stated that negative emotional states (i.e., math anxiety) affect complex arithmetic performance more than simple arithmetic performance (Ashcraft, 1995; Devine et al., 2012).

Stressful situations can also be induced by manipulating the context in which the problem is presented, such as punishing poor performance with social consequences, in order to interfere with cognition (Beilock and Carr, 2001, 2005). In other words, taxing people's executive resources by increasing anxiety due to fear of negative consequences resulted in less efficient strategy use, and consequently poorer arithmetic performance.

In this review, we summarize the state of research about the relationship between cognitive stress and strategic behavior used to solve arithmetic tasks. The synthesis has been complicated by the use of different terms according to the specific line of research. In particular, studies on stress and math-related emotions each look at slightly different domains and consequently refer to them with slightly different terms. For example, “stress,” “negative emotions” and “anxiety” are labels which, in the mathematical research field, have often been used to describe similar states of mind that potentially can interfere with the execution of mathematical tasks (Stipek and Gralinski, 1991; Galla and Wood, 2012; Brunyé et al., 2013). In the first part of this article we present and discuss different conceptual and methodological approaches related to manipulating task complexity, focusing in particular on the different ways to trigger cognitive stress. In the second part we pay particular attention to time pressure manipulation, by highlighting its effect on strategy choice and cognitive processes and its relationship with emotional aspects, such as math anxiety.

### Electronic searches and selection of studies

An electronic search was conducted on principal databases (PsychINFO, Web of Science, PubMed, EBSCO, Scopus) for English published articles. No date restriction was used, and the keywords were: time pressure/time constraint/time limit/time deadline; strategy/strategy selection/strategy choice/strategy efficiency; problem solving/arithmetic/math/mathematics/calculation; emotional factors/cognitive stress/anxiety/math anxiety. The search has been done with the following combinations of terms: (time pressure OR time limit OR time constraint OR time deadline) AND (arithmetic OR math^*^ OR calculation OR problem solving) AND (strateg^*^) AND (emot^*^ OR stress OR anxiety). We used the wildcard ^*^ where alternative words like “strategic”/“strategy” or “emotion”/“emotional” might arise. As allowed by each database, the terms have been explored mainly in the title, abstract and keywords, and when possible through the entire full text.

Research was included in this review by following these inclusion criteria. First, since studies on this topic vary in their methodological design, we included those studies which clearly stated the manipulation of the execution time during the main task; second, if they considered how the time constraint affected participants' strategic behavior and consequently their results; and finally, if they have quantitatively measured emotional aspects related to math tasks. Studies aiming to highlight gender differences or that revealed different sample sizes according to gender were excluded, as well as research primarily focused on neuroimaging effects.

We considered papers published before December 2016. The initial inspection was independently completed by two reviewers (SC and EC): the electronic search identified 2,534 papers which matched the search terms. After deleting duplication 352 studies were selected. Titles and abstracts of the studies retrieved were then screened by two reviewers to identify studies that potentially met the criteria outlined above. The full text of the 129 remaining potentially eligible studies were then retrieved and independently assessed for eligibility by two reviewers: any disagreement between the two reviewers was resolved through discussion with a third reviewer (DS): 56 papers remained at this stage.

It is important to note that the inclusion criteria we used in the papers selection led us to exclude all the studies which applied a time limit constraint due to the experimental setting, i.e., studies which did not actually impose a direct time limit on the performance, but instead looked at results by setting a *post-hoc* deadline for responses (e.g., ERP studies for time course analysis of arithmetic information processing; Rosenberg-Lee et al., 2009; Hinault and Lemaire, 2016). A total of other 21 studies were excluded at this stage.

**Figure 1:**
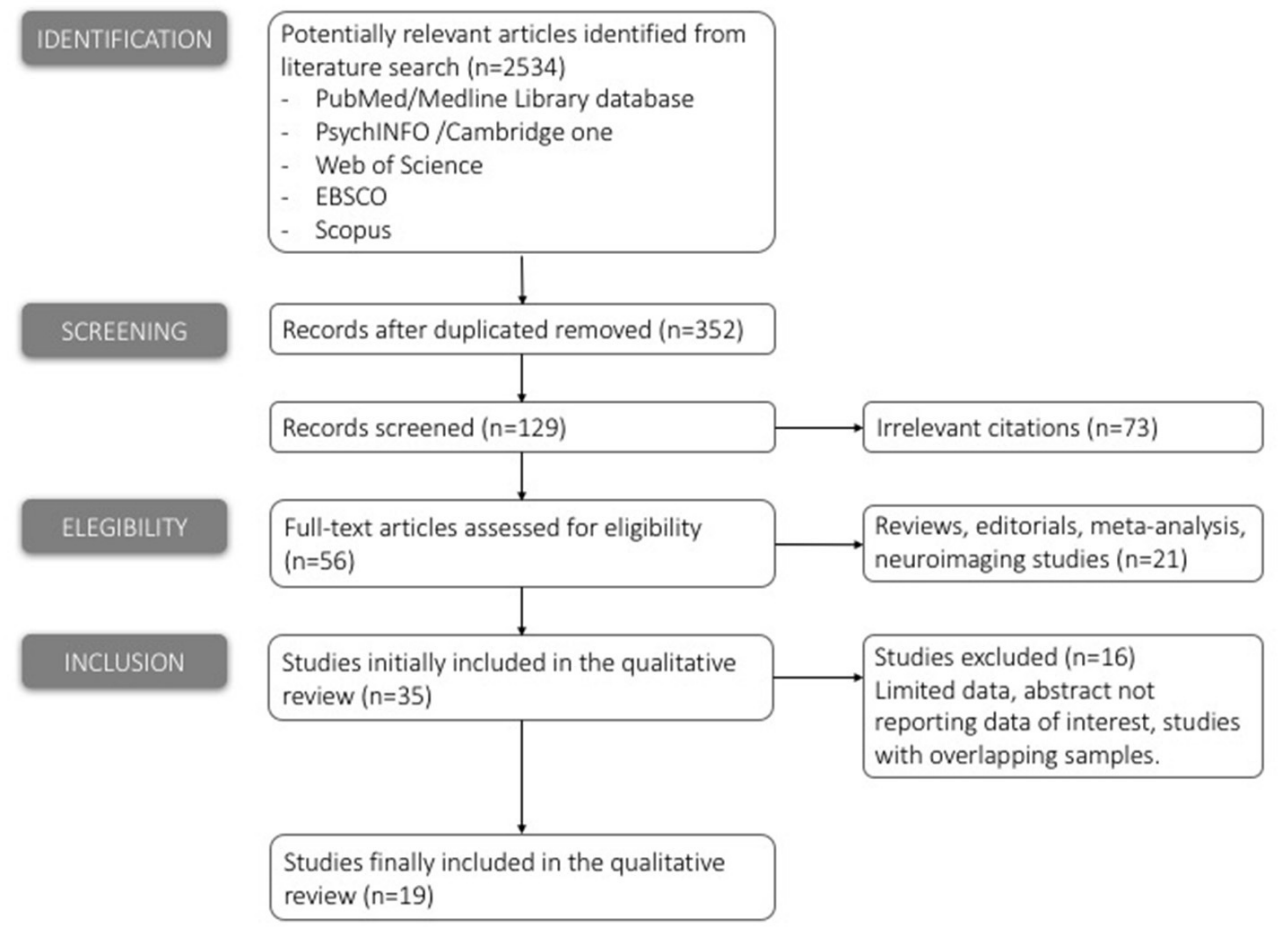
Flow chart summarizing the electronic search.

Finally, 35 studies attained the eligibility criteria. A pre-prepared Excel spreadsheet was used to record extracted data from the included studies for assessment of study quality and data synthesis. Study quality consisted of a risk of bias assessment: quality of individual studies was evaluated in terms of sample size and type (and number) of tasks reported for each domain (time limits/mathematical tasks/emotional factors). Where there was concern over the methodological quality of any studies, sensitivity analyses were conducted: only 19 met the selection criteria and were included in the present review (see Figure 1). Once the target articles had been agreed, the two independent reviewers (SC and EC), by using a customized scheme, extracted the relevant data. Besides participants' age, information about (1) experimental design and timing, including the type of arithmetical task and relative strategies, (2) emotional aspects and (3) the type of cognitive processes investigated (if present), were collected. We also noted whether studies included children (6–12 years), adolescents (13–17 years) or adults (18 years and above).

Regarding the experimental designs, we preferred not to use any restrictive classification of the mathematical tasks reported, but due to high heterogeneity, we report a description of the task itself. Specifically related to the “pressure” aspect, we tried to classify how pressure was induced in the experimental design, in particular whether (i) it was provided by inducing a time limit or by means of other manipulations (e.g., monetary incentives), (ii) in the case of time limits, we considered extensively the experimental methodology (e.g., number of conditions, time limit applied to stimuli presentations or response window).

### Structure of the review

The main goal of this review was to shed light on how stress induction, particularly time pressure manipulations, can interfere with or modulate strategy selection in arithmetical tasks. Additionally, we were also interested in whether this association could be moderated by emotional and cognitive factors.

The electronic search and the subsequent screening phases highlighted how stress induction or time pressure manipulation have been differentially implemented to trigger cognitive stress during math task execution. Indeed, among the 19 relevant articles identified, 8 papers applied social constraints to induce pressure. Eleven further studies implemented a time pressure manipulation to induce pressure.

**Figure 2:**
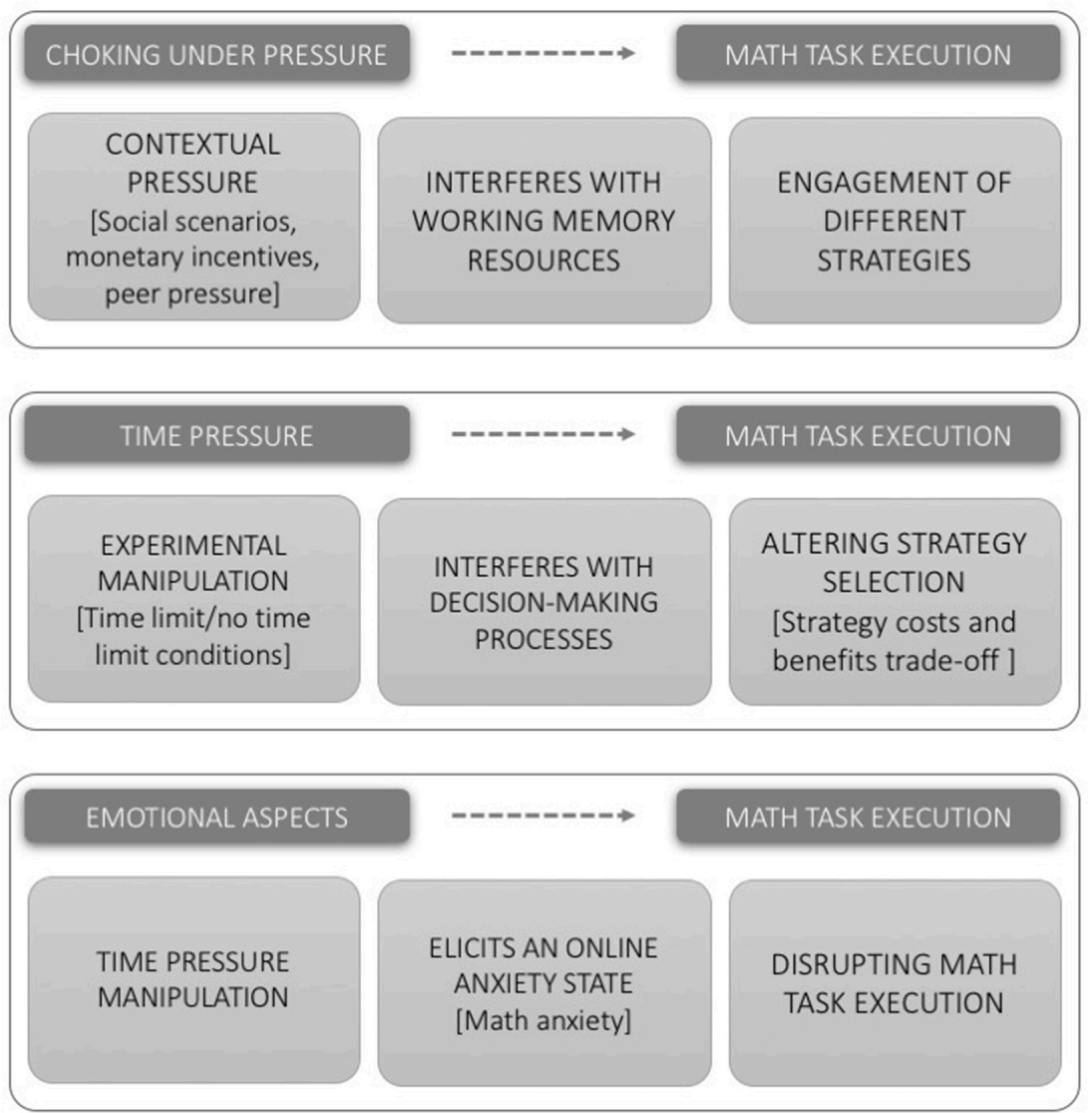
Graphic summary of the three principal mechanisms/relationships discussed in different sections of the manuscript.

In the subsequent section of this article (Social Stress Induction and Choking under Pressure Phenomenon), we first consider those papers which induce stress and “choking under pressure” via social manipulations which do not limit time available to solve tasks. In the next section (Time Pressure in Math: Strategy Selection), we consider tasks which engaged a time pressure manipulation. These two types of pressure manipulation are different in nature for a fundamental reason: regardless of “choking under pressure,” time pressure manipulations may render a previous strategy useless due to complexity. With only limited time available, rapid heuristic strategies can become optimal simply due to their speed. Finally, in Section Emotional Aspects and Stress Manipulation, we consider those studies which actually measure affective factors (such as mathematics anxiety). There is a paucity of such studies, making firm conclusions challenging: however, they are of importance in determining the role of pressure in math tasks (see Figure 2 for a graphic summary).

The outcomes of the present review only partially resolve our initial questions and they can be understood more as open questions than evidence of clear associations. Each section concludes by raising a series of aspects in which this field of research may move forward.

### Social stress induction and choking under pressure phenomenon

As noted, cognitive stress may influence cognitive resources (Mazzoni and Cornoldi, 1993; Barrouillet et al., 2007) and consequently affects strategic behavior in different domains, such as mathematical learning, which often involves high-pressure tests. Especially in math, where stress and anxiety are common and there is a strong desire to perform well, people may fail to perform at their best level, despite having the required skill (Benny and Banks, 2015).

Surveying the literature regarding stress-induced performance in mathematical domain, one of the main topics referred to is the “*choking under pressure*” phenomenon. Choking, or worse performance than expected for one's level of ability, tends to happen in situations involving performance pressure (Baumeister, 1984; Lewis and Linder, 1997; Beilock and Carr, 2001). Table 1 summarizes this research.

**Table 1:** Studies investigating cognitive stress and “choking under pressure” phenomenon.

	**Authors (Year)**	**Age (*N*)**	**Math task**	**Pressure**	**Strategies**	**Emotional aspects**	**Cognitive processes**
1.	Beilock et al., 2004	Undergraduate students (Study 1: 40 and 40)	Modular arithmetic task (MA, verification task)	Monetary incentives, peer pressure, social evaluation	NA	State-Trait Anxiety Inventory (STAI)	NA
2.	Beilock and Carr, 2005	Undergraduate students (93)	Modular arithmetic task (MA, verification task) and a paper-and- pencil division, subtraction and multiplication task	Monetary incentives, peer pressure, social evaluation	NA	NA	Automated Operation Span task and Reading Span task
3.	Beilock and DeCaro, 2007	Undergraduate students (Study 1: 44 and 48; Study 2: 46 and 45)	Study 1: modular arithmetic task (MA, verification task). Study 2: water jug problems	Monetary incentives, peer pressure, social evaluation	Written self-report (1) Rule-based algorithm that involved a series of step-by-step computations (2) Estimation or guessing based on previous associations (3) No sense	State-Trait Anxiety Inventory (STAI)	Automated Operation Span task and Reading Span task
4.	Beilock, 2008	NA	Modular arithmetic task (MA, verification task)	Monetary incentives, peer pressure, social evaluation	NA	NA	NA
5.	DeCaro et al., 2010	College students (78)	Modular arithmetic task (MA, verification task): vertical and horizontal presentation	Monetary incentives, peer pressure, social evaluation	NA	Non standardized retrospective verbal report	Talk-aloud/No-talk aloud conditions
6.	Wang and Shah, 2014	Third and fourth graders (53)	3-digit mental addition problems	Videotaped and external evaluation performance	NA	NA	Automated Operation Span task
7.	Benny and Banks, 2015	Undergraduate students (60)	Modular arithmetic task (MA, verification task)	Monetary incentives, videotaped and external evaluation performance	NA	State-Trait Anxiety Inventory (STAI); Need Cognition Scale (NFC); Thought Probes during the MA task	Automated Operation Span task and Reading Span task
8.	Sattizahn et al., 2016	Adults (85)	Modular arithmetic task (MA, verification task)	Monetary incentives, peer pressure, social evaluation	NA	State-Trait Anxiety Inventory (STAI)	Arrow-based flanker task; Automated Operation Span task and Reading Span task

*N, participants number; NA, not applicable*.

The main hypothesis of this research field is that contextual pressure interferes with limited WM resources (Miyake and Shah, 1999; Jonides et al., 2008). According to this perspective, pressure is assumed to cause worrying off-task thoughts that result in overload of WM already engaged in the math task, similarly to math anxiety. Beilock and colleagues (Beilock et al., 2004; Beilock and Carr, 2005; Beilock and DeCaro, 2007; Beilock, 2008) investigated how individual differences in WM capacity relate to high and low pressure conditions defined by different social scenarios, such as monetary incentives or peer pressure, while subjects solve modular arithmetic problems (a complex task that can be solved by computation or estimation strategies). They found that participants with higher WM capacity were more disturbed by the pressure constraint than the subjects with less WM capacity, leading to a more significant lowering of arithmetic performance in those with higher WM capacity.

In subsequent research, Beilock and DeCaro (2007) tried to disentangle the issue of which strategy was engaged by analyzing the availability of WM resources and task complexity within a single study. In all their studies the authors manipulated the pressure condition by prompting commonly experienced pressures in everyday life, such as monetary reward, peer pressure, and social evaluation. The authors found that the difference in terms of performance between high- and low-WM participants was linked to the strategy selected to solve the problems: because high WM individuals typically use more WM-intensive strategies than low WM individuals, their strategies were affected more strongly by performance pressure (DeCaro et al., 2010). Similar results have also been reported in primary school children who had to solve mental arithmetic tasks (Wang and Shah, 2014). This data partially replicated previous findings but also added something new: the presence of a pressure condition (induced by simulating the recording of an evaluation video) affected children' math performance depending on their own cognitive resources and the specific task difficulty. In this study, high-pressure scenarios led to more errors when high WM children had to solve normal carry problems, whereas low WM children choked under pressure when they had to solve hidden carry problems, i.e., problems that can only be solved successfully through computational strategies. No such distinction emerged from individual differences in WM with simpler no-carry problems, regardless of the pressure. This effect seems to be strictly connected to the strategy selected and applied to solve the problem. According to the authors, these problems could be solved with two main strategies: computational strategies, that are WM demanding, or more heuristic and estimation strategies. Children' decision to use computational strategies or easier heuristics to solve problems is dependent on both task complexity and a subject's available working memory resources, influenced by the presence of pressure. In the pressure condition, low WM children were not able to rely on computationally demanding strategies to solve for hidden carry problems, so were forced to use the less demanding and also less accurate heuristic strategies. These results show that different strategies are optimal depending on the mental state induced by the problem context. When a factor reducing WM capacity is in place, students may be better to use less accurate but less WM-intensive strategies. On the other hand, when WM capacity is higher, more WM-intensive and higher accuracy strategies are more worthwhile (Beilock and DeCaro, 2007; Benny and Banks, 2015; DeCaro et al., 2016).

Recently, Sattizahn et al. (2016) replicated previous results demonstrating that adults with high WM resources performed significantly worse due to experiencing high pressure. They also tested individuals' variability in attentional control processes, finding that differences in attentional control influenced the effect of a pressure situation. Those with low attentional control suffered decreased performance under pressure, whereas those with high attentional control did not. This likely reflects that some individuals are able to prevent the interfering effect of pressure on their performance, whereas other with lower attentional control are not able to do this.

Although the above studies seem to answer a distinctive question (how can pressure influence math performance?), they differ in so many aspects, making it difficult to reach a certain answer. For example, all the above research aimed to analyze the effect of pressure on math, but none of them implemented a quantifiable manipulation of pressure with controlled conditions. Instead, they engaged social scenarios which could differentially affect each participant. Similarly, the mathematical tasks and cognitive measures considered vary widely across studies. In order to clarify findings in this domain it is important to systematically consider the role of task difficulty, the nature of the tasks themselves (those that allow for multiple strategies vs. those that do not), and the type of pressure manipulation used in the different studies. This leads to another question: how else can cognitive stress be induced in the math domain?

### Time pressure in math: strategy selection

A large amount of literature from the decision-making field suggests that manipulating the time limit/pressure associated with a task has a very strong effect on the stressful nature of that particular task (e.g., Kerstholt, 1994; Ordóñez and Benson, 1997; Huber and Kunz, 2007; Young et al., 2012; Cone and Rand, 2014; Byrne et al., 2015). However, in the mathematical cognition field few studies have considered a time deadline as an important interference source and explicitly manipulated it by inserting in the experimental design at least two separate conditions; one with and one without a time limit.

One of the first studies on this topic attempted to simulate a computational model of automated and controlled processing during the execution of addition problems in trinary notation: Richardson and Hunt (1985) addressed the issue of how “problem solving sometimes takes place under severe real time constraints” by inserting a series of interruptions during stimulus presentation. Subsequent research has manipulated the time limit using more controlled experimental designs, across a variety of mathematical tasks, such as numerosity judgment (Luwel and Verschaffel, 2003) arithmetical problems (Kellogg et al., 1999; Campbell and Austin, 2002), probabilistic and proportional reasoning (Gillard et al., 2009; Agus et al., 2015), and algebraic concepts (McNeil et al., 2010; Chesney et al., 2013). A summary of the studies which implemented a time pressure manipulation is listed in Table 2.

**Table 2:** Studies investigating strategy selection (positions 1–8) and emotional aspects (position 9–11) with time condition manipulation.

	**Authors (Year)**	**Age (*N*)**	**Math task**	**Time pressure**	**Strategies**	**Emotional aspects**	**Cognitive processes**
1.	Richardson and Hunt, 1985	College students (4)	Addition problems in trinary notation	From time to time the arithmetic task was interrupted and then resumed	NA	NA	NA
2.	Schunn et al., 1997	Graduates and undergraduates students (25)	Arithmetic problems (half multiplication, and half invented operator)	Time constraints linked to the strategy choose (2 s vs. 20 s)	Participants chose between retrieval and calculation strategy	NA	NA
3.	Campbell and Austin, 2002	Undergraduate students (48)	Simple addition problems	Time limit: 750 ms for the fast deadline; 2,500 ms for the slow deadline	After each problem participants chose one among 4 alternatives: Transformation, Counting, Remember, Other	NA	NA
4.	Luwel and Verschaffel, 2003	Sixth-grade children (81)	Numerosity judgment task	Time pressure: manipulation of the stimuli presentation time (3 conditions: severe (5 s), moderate (10 s) and low (20 s) time pressure condition)	The three strategies (addition, subtraction and estimation) applied were identified by fitting the individual response-time pattern	NA	NA
5.	Gillard et al., 2009	Study 1: undergraduate students (167)	Proportional and non-proportional (additive) word problems	Time limit: 17 s for the fast deadline; no time limit for the control condition	NA	NA	Study 2: Dot memory task as secondary task
6.	McNeil et al., 2010	Undergraduate students (184)	Addition facts and equations (*a*+*b*+*c* = *d*+__)	Study 1–3: timed presentation of the equations (1000–1500ms); study 4: untimed presentation	Add-all and add-to-Equal Sign strategies (indirectly assessed)	NA	NA
7.	Chesney et al., 2013	Undergraduate students (64)	Equations (a+b+c = d+__)	Half trials were timed (half were untimed): equation remained on the screen for 2 s	Responses were coded as reflecting a particular strategy: Correct; Add-all; Add-to-equal; Add-two; Carry; Repeat; Other	NA	NA
8.	Agus et al., 2015	Undergraduate students (676)	Probabilistic reasoning	Time limit condition: 30 min	NA	Statistical Anxiety Scale (SAS)	Visuo-spatial and numerical scales (Primary Mental Abilities—PMA); Attitudes Toward Statistics (SATS-28)
9.	Plass and Hill, 1986	Third- and fourth-grade children (155)	Test booklets with 15 arithmetic word problems with multiple-choice answers	Time-pressure condition: when 2 of the children in any group had finished, the experimenter stopped the time	NA	Test Anxiety Scale for Children (TASC): Lie Scale for Children (LSC)	NA
10.	Kellogg et al., 1999	Undergraduate students (30)	10-page arithmetic questionnaires (simple and complex problems)	Timed condition: 70% of the mean response time for each page	NA	Abbreviated Math Anxiety Rating Scale (sMARS)	NA
11.	Tsui and Mazzocco, 2006	Sixth grade gifted children (36)	Math calculations involving fractions, decimals, calculus, and trigonometry (similar to the calculation test, Woodcock-Johnson III)	Timed condition: 10-min time limit to finish the math calculation task	NA	The Mathematics Anxiety Rating Scale Elementary (MARS-E); the Screen for Child Anxiety Related Emotional Disorders (SCARED); the Multidimensional Perfectionism Scale (MPS)	NA

As in the decision-making domain, time pressure in mathematics has been manipulated primarily by limiting available time for each decision or choice. Several studies have shown that time pressure interferes with decision-making by altering strategy selection. The presence of a time constraint in any math or problem-solving situation can affect performance: the presence of time limits could either encourage students' engagement with the task or increase choice of the wrong strategy for that task (Beilock and DeCaro, 2007; Rieskamp and Hoffrage, 2008). Leaving an open time window to complete a task enables participants to get a greater amount of information by focusing attention on important task features, which results in the best strategy selection (Payne et al., 1988; Siegler and Lemaire, 1997; Rieskamp and Hoffrage, 2008; Heinze et al., 2009). According to this viewpoint, Gillard et al. (2009) manipulated time pressure by reducing the solution time or introducing a concurrent task, leading to a self-imposed time constraint to complete the main task (see for Rieskamp and Hoffrage, 2008). The authors compared heuristic (which engage faster and automatic processes) and analytic processes during the execution of proportional and non-proportional problems in university students in two different experiments. They found that limiting students' resources, by reducing their response time or loading their working memory system, resulted in an increase in the wrong choice of heuristic proportional solution strategy.

Thus, time pressure is one factor that influencing which strategy people select to deal with a particular math problem situation (Young et al., 2012; Alison et al., 2013). McNeil and colleagues investigated how time pressure can influence the strategies used by both adults and children to solve equation problems. They found that under time pressure, university students used the same typical arithmetic strategies applied by children to solve mathematical equivalence problems, demonstrating how people can shift from more complex to simpler strategies when they are under pressured conditions (Mcneil and Alibali, 2005; McNeil et al., 2010; Chesney et al., 2013). Similarly, Campbell and Austin (2002) used a time limit manipulation when adults were solving simple addition problems in order to alter their strategy choice. The author imposed a fast deadline (750 ms) to force adult participants to use a retrieval strategy, and a slower one (2,500 ms) to elicit use of procedural strategy (e.g., counting or transformation). Results indicated adaptive strategic behavior (Siegler and Lemaire, 1997) which was influenced by problem features. The fast deadline resulted in a small effect on retrieval strategy usage for smaller problems, where fact retrieval was used under both conditions. On the other hand, for larger problems, participants report decreased use of procedural strategies under the faster condition. The time pressure imposed by the fast deadline adaptively modified the participants' selection strategy increasing the attempts to solve larger problems by a retrieval strategy. For large problems under the time pressure condition, retrieval strategy was both faster and more accurate than a multistep procedure, making this an example of adaptive strategy selection.

A similar pattern of results has been found by Luwel and Verschaffel (2003) testing the estimation strategies of sixth graders under three different time pressure conditions. In this study, participants were asked to determine the number of filled blocks in a 10 × 10 grid as accurately and fast as possible, according to time pressure conditions, operated by introducing three temporal windows of stimulus presentation (5, 10, or 20 s). To accomplish the task, participants would engage three possible strategies, identified in prior studies, each of which is expected to elicit a specific pattern of response times (and deviation scores) as a function of the number of blocks present (Luwel et al., 2000, 2001). Analysis of the results revealed that children's performance was also affected by increasing time pressure on strategy repertoire, relative frequency of strategy use and efficiency of strategy execution, indicating that even at a young age children seem able to adapt their strategy use to the external task demands, in terms of coping with the given time restrictions (for a different pattern of results, see Schunn et al., 1997).

The overall results highlight that time pressure adjusts the strategy decision process generating a sort of strategy costs and benefits trade-off: when the available time is short and the task complexity is substantial (i.e., Campbell and Austin, 2002), strategies that can be applied rapidly represent the more appropriate choice. On the other hand, leaving an open time window to complete a task enables participants to get a greater amount of information by using a slower but more accurate strategy (Payne et al., 1988; Siegler and Lemaire, 1997; Rieskamp and Hoffrage, 2008; Heinze et al., 2009). A reduction in execution time allowed to solve a mathematical task can lead to a decrease of information that can be collected. The importance of reaction time analysis in the study of mathematical processes is widely acknowledged, above all in the domain of strategy selection (DeCaro et al., 2016). For example, it is worth noting that switching between complex and shortcut strategies incurs a cost to reaction time (e.g., Luwel et al., 2009; Schillemans et al., 2009; Lemaire and Lecacheur, 2010), but this can be directly tested only when the experimental paradigm requires there to be no time limit in place.

### Emotional aspects and stress manipulation

The negative emotional state and discomfort felt during performance of mathematical tasks is commonly referred to as math anxiety (Hembree, 1990; Ma, 1999; Ma and Xu, 2004). The current consensus is that math anxiety is negatively correlated with mathematical performance (Ashcraft, 2002; Devine et al., 2012; Carey et al., 2016; Hill et al., 2016). Most of the research measures math anxiety levels through self-report questionnaires detecting a sort of “offline” measure rather than testing anxiety levels while solving math problems (e.g., Trezise and Reeve, 2014, 2015). This assessment of math anxiety implicitly leads one to assume that it is an enduring anxiety (trait) rather than an anxiety state experienced whilst solving particular problems. On the other hand, manipulating time pressure during a mathematical task might elicit an online anxiety state that allows accurate analysis of how anxiety can disrupt or interfere with arithmetic task execution.

Although it seems to be widely recognized that providing a reasonable time for the accomplishment of a math test should be effective in reducing at least some of the disadvantage experienced by math anxious subjects (Faust et al., 1996; Ashcraft and Kirk, 2001), surprisingly little research directly includes a time condition manipulation. To our knowledge, only three studies involved a clear time pressure manipulation for testing the effect of math anxiety on mathematical tasks (see Table 2, studies numbered 9–11).

Plass and Hill (1986) analyzed the relation between problem solving ability, test anxiety and gender differences in 155 third- and fourth-grade primary school children. The sample was divided into three groups (low, middle, and high test-anxiety) either under time or no-time pressure conditions. Under time pressure, high- and middle-anxiety children performed worse than low-anxiety children of both genders. The removal of time pressure strongly improved performance for anxious boys but not for girls.

Tsui and Mazzocco (2006) examined the effects of math anxiety on math performance, under timed and untimed testing conditions in 36 sixth grade primary school children. They found a general pattern of reduced accuracy in math performance under the timed testing condition. This was influenced by participants' math anxiety level: higher anxiety children performed equally under timed and untimed testing conditions. Conversely, lower anxiety children had decreased math performance under timed condition. Although there was no main effect of gender on timed vs. untimed math performance, boys were equally accurate on timed and untimed testing, by contrast, girls showed a discrepancy in accuracy in favor of untimed conditions. They explain this pattern of results in terms of facilitating anxiety: according to the authors, the performance of gifted children with high math anxiety did not drop under time pressure because of math anxiety canceling out the negative effect of time pressure; effect that was present for lower math anxiety children.

These outcomes do not converge with Kellogg et al. (1999) who tested 30 undergraduate university students divided into three different groups according their math anxiety level. Participants were asked to solve a series of arithmetical tasks in both a timed and untimed condition. Kellogg et al. (1999) did not observe any difference between high and low anxiety individuals, although the timing manipulation negatively affected the arithmetic performance of both groups. The authors stated that time pressure manipulation had an additive effect with anxiety on arithmetic performance. Consequently, although “worry” may adversely affect the performance of highly anxious individuals (Eysenck and Calvo, 1992), it does not appear that the level of worry is differentially related to the amount of time assigned to perform a mathematical task. In other words, they concluded that time pressure was not a contributor to the worrisome thoughts that occupy individuals with high math anxiety during arithmetic testing.

In sum, there is some evidence that math anxiety interacts with timed or high-stakes conditions to cause a further performance decrement than usual. However, due to the paucity and heterogeneity of research both in terms of sample and tasks considered, these results do not allow us to conclude that increasing time pressure has a differential effect depending on math anxiety. Similarly, assuming causal relations between time pressure and inducing math anxiety currently does not have evidential support.

## General conclusions

This literature overview of the past 30 years focuses on the effect of stress and/or time pressure on math proficiency. It has revealed that, generally speaking, pressure has a great influence on both strategic and emotional aspects of task execution.

Research on choking and excelling under pressure has focused on tasks that demand many cognitive resources, especially working memory (Miyake and Shah, 1999; Jonides et al., 2008). Similarly, some research on math anxiety suggest that trait anxiety also reduces effective working memory capacity (Ashcraft and Kirk, 2001; Ashcraft and Krause, 2007). This suggests that anxiety impairs performance by overloading working memory. Specifically, pressure is expected to lead to worry, concern and other distracting thoughts about performance, which consume working memory resources (Beilock and Carr, 2005).

It has been widely assumed that people are equipped with a range of cognitive strategies which they adaptively select and apply according to the specific task and situation. Within this framework, pressure represents one factor that can influence which strategy people select to deal with a particular situation. Relatively little research has focused on the impact of time pressure on strategy selection in mathematics, principally aiming to show how time pressure interferes with the decision process in terms of strategy selection in mathematical domain. Results seem to suggest that time pressure generally acts as a stressor, causing suboptimal strategy selection. However, the causal mechanism of this is still unclear. It is not clear whether time pressure interferes with strategy *selection* or whether it simply renders the optimal strategy impossible, due to an overload of working memory resources.

Further research is needed to address this question, taking into account the huge variability of execution time linked to task type (e.g., simple vs. complex calculation or even verification vs. production task; Ashcraft, 1995; Rousselle and Noël, 2008). For example, the answer modality of a math task (verification vs. production) can strongly influence and drive the solution process itself, and consequently the strategies applied to solve the task. Sometimes, experimenters chose to adopt a verification mode (i.e., namely a choice among a series of alternatives) compared to a production task (i.e., participant is asked to produce/give the right answer) for time limit issues linked to the experimental setting or data analyses (e.g., ERP study for time course analysis of arithmetic information processing; time limit set up only during data analysis to identify outliers or specific retrieval answers). Within this perspective, the studies summarized in the “*choking under pressure*” section mainly reported verification tasks, conversely, the experiments listed in the other subsequent sections were often production tasks, making it harder to provide general conclusions based on this methodological aspect.

Similar considerations can be drawn regarding the relationship between time pressure and emotional aspects within the mathematical learning framework, leaving space for several open questions. Among them is whether time pressure can be always considered as a negative factor in terms of proficiency and math anxiety. To date the literature does not clearly answer this issue. Decreased performance under time pressure is not consistently observed in high or low math anxiety individuals. Previous problem-solving studies suggest that time constraints inhibit creative thinking; but more recent research indicates that time constraints can sometimes prove beneficial (Medeiros et al., 2014). An alternative explanation of this inconsistent pattern may be found in the social pressure literature by considering where individuals focus their attention during the performance. It may be important whether attention is directed on the process of performance or to the outcome of performance: these situational aspects of the attentional system may affect results. Pressure does not simply cause a reduction in executive resources; it changes one's motivational state, leading to failure or success with different types of tasks due to the availability of attentional resources during performance (see e.g., Markman et al., 2006; Worthy et al., 2009).

To sum, the present review demonstrates the need for a broader view of the effects of time pressure on math performance. Future research should systematically examine the effects of time pressure on math performance and strategy selection to develop a fuller framework of phenomena that drive choking or excelling under pressure.

## Author contributions

SC and DS developed the study concept. Literature review was conducted by SC and EC. SC and IM drafted the manuscript. DS provided critical revisions. All authors approved the final version of the manuscript for submission.

### Conflict of interest statement

The authors declare that the research was conducted in the absence of any commercial or financial relationships that could be construed as a potential conflict of interest.
